# NADPH Oxidase Deficiency: A Multisystem Approach

**DOI:** 10.1155/2017/4590127

**Published:** 2017-12-21

**Authors:** Giuliana Giardino, Maria Pia Cicalese, Ottavia Delmonte, Maddalena Migliavacca, Boaz Palterer, Lorenzo Loffredo, Emilia Cirillo, Vera Gallo, Francesco Violi, Claudio Pignata

**Affiliations:** ^1^Department of Translational Medical Sciences, University of Naples Federico II, Naples, Italy; ^2^San Raffaele Telethon Institute for Gene Therapy (SR-TIGET), San Raffaele Scientific Institute, Milan, Italy; ^3^San Raffaele Telethon Institute for Gene Therapy (SR-TIGET), Pediatric Immunohematology and Bone Marrow Transplantation Unit, San Raffaele Scientific Institute, Milan, Italy; ^4^Division of Immunology, Boston Children's Hospital, Boston, MA 02115, USA; ^5^Department of Experimental and Clinical Medicine, University of Florence, Florence, Italy; ^6^Department of Internal Medicine and Medical Specialties, Sapienza University of Rome, Rome, Italy

## Abstract

The immune system is a complex system able to recognize a wide variety of host agents, through different biological processes. For example, controlled changes in the redox state are able to start different pathways in immune cells and are involved in the killing of microbes. The generation and release of ROS in the form of an “oxidative burst” represent the pivotal mechanism by which phagocytic cells are able to destroy pathogens. On the other hand, impaired oxidative balance is also implicated in the pathogenesis of inflammatory complications, which may affect the function of many body systems. NADPH oxidase (NOX) plays a pivotal role in the production of ROS, and the defect of its different subunits leads to the development of chronic granulomatous disease (CGD). The defect of the different NOX subunits in CGD affects different organs. In this context, this review will be focused on the description of the effect of NOX2 deficiency in different body systems. Moreover, we will also focus our attention on the novel insight in the pathogenesis of immunodeficiency and inflammation-related manifestations and on the protective role of NOX2 deficiency against the development of atherosclerosis.

## 1. Introduction

Nicotinamide adenine dinucleotide phosphate (NAPDH) oxidase (NOX) is a multimeric complex composed of enzymes of the NOX family [1]. NOX2 is a transmembrane protein firstly identified in phagocytic cells (e.g., neutrophils, eosinophils, and macrophages) [2] and dendritic cells [3–6]. It plays a crucial role in antimicrobial host defence and inflammation. NOX2 deficiency leads to the development of chronic granulomatous disease (CGD), a primary immunodeficiency characterized by life-threatening bacterial and fungal infections [7]. In addition to recurrent infections, patients with CGD also suffer from impaired inflammatory responses leading to the development of Crohn's-like inflammatory bowel disease (IBD) [8, 9] and other inflammatory complications [7, 10, 11]. Recently, increased reactive oxygen species- (ROS-) independent inflammasome activation and impaired autophagy, resulting in increased IL-1b release, have been shown in NOX2-deficient phagocytes [12, 13]. The treatment with the IL-1b receptor inhibitor (anakinra) resulted in decreasing the activation of inflammasome and in restoring autophagy in mice with CGD in vitro and in vivo. While the treatment seems to exert the same effect in human cells in vitro, the treatment in vivo is associated with contrasting results in humans [14, 15]. Rapamycin, a potent mammalian target of rapamycin (mTOR) inhibitor and autophagy inducer, has been shown to be able to restore autophagy and to regulate inflammasome activation in patients with CGD, unravelling new therapeutic opportunities for the treatment of inflammatory manifestations in CGD [16, 17]. Apart from its well-characterized role in the phagocyte function, NOX2 seems to be also implicated in the function of other different organs. In fact, *NOX2* is also expressed in endothelial cells [18], cardiomyocytes [19], hematopoietic stem cells (HSC) [20, 21], and platelets [22] and its upregulation has been shown to be involved in neurodegenerative disease [23], neoplasms [24], and cardiovascular diseases [25–28]. Recent studies suggest that NOX2 activation is also involved in the development of atherosclerosis. In patients with CGD and in heterozygous carriers, the reduction of the ROS production is associated with increased levels of flow-mediated artery dilatation, thus implying a lower risk of developing atherosclerotic manifestations [29–32].

Other different NOX isoforms are implicated in diverse physiological functions, and they can be identified in numerous cell types [33]. Evidence suggest that NOX/DUOX enzymes are implicated in a number of biological processes including host defence, regulation of the vascular tone, hormone synthesis, fertilization, cell proliferation and differentiation, and formation of the extracellular matrix [33, 34].

In this review, we will summarize the effect of NOX2 deficiency in different body systems, focusing our attention on the novel insight in the pathogenesis of immunodeficiency and inflammation-related manifestations and on the protective role of NOX2 deficiency against the development of atherosclerosis. We will also focus on the novel insight in the definition of the clinical phenotype in heterozygous and female carriers and on the efficacy of novel and traditional potential therapeutic approach. Moreover, we will summarize the role of other NOX/DUOX enzymes in the function of different body systems and in the development of disease in humans.

## 2. NADPH Oxidase: Structure and Function

**Figure 1:**
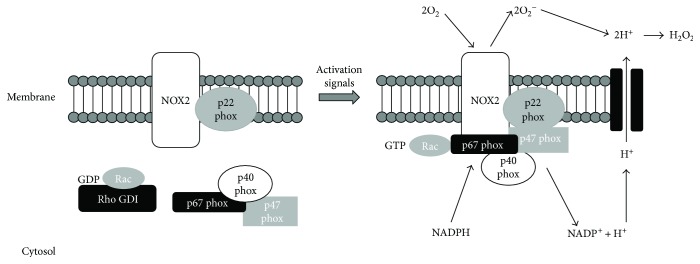
Schematic representation of the inactive and active forms of the NADPH oxidase complex. The complex consists of six subunits. NOX2 and p22phox are associated to form a heterodimer bound to the plasma membrane in both the inactive and the active forms. In resting conditions, p47phox, p67phox, p40phox, and the G-protein Rac are located in the cytosol. In the active form, the cytosolic subunits associate with the membrane-bound NOX2/p22phox heterodimer. The active NADPH oxidase generates superoxide (O_2_^−^) by transferring electrons from NADPH inside the cell across the membrane and coupling them to molecular oxygen.

NOX plays a pivotal role in the production of ROS and, in particular, of the superoxide anion (O_2_^−^) at the expense of NADPH. Seven genes have been identified encoding for NOX isoforms: *NOX1* to *NOX5*, including *CYBB* gene encoding for NOX2, *DUOX1*, and *DUOX2* [33, 35]. The different NOX isoforms in humans are involved in a wide range of cellular processes, including apoptosis, host defence, cellular signal transduction, oxygen sensing, and angiogenesis. Although NOX is ubiquitously expressed, the distribution of different isoforms is cell or tissue specific, allowing each NOX a distinct physiological and pathological function. All the members of the NOX family have conserved structural properties which are responsible for their catalytic activity. In particular, each homolog is composed of six or seven transmembrane domains, with two hemes in the N-terminal region containing histidine residues and a NADPH-binding site in the cytoplasmic C-terminal. Moreover, all the isoforms form multimeric complexes characterized by the presence of a core catalytic subunit and up to five regulatory subunits [36]. The regulatory subunits have essential biological roles. In particular, the regulatory subunits p22phox, DUOX activator 1 (DUOXA1), and DUOXA2 are involved in the maturation and expression of the NOX/DUOX subunits in cell membranes; p67phox and NOX activator 1 (NOXA1) are essential for enzyme activation; p47phox, NOX organizer 1 (NOXO1) and p40phox have a role in spatial organization of the complex. Some NADPH oxidase isoforms, including NOX2, also rely on a small GTPase (RAC1 or RAC2) for their activation [37]. The NOX2 complex consists of six subunits, including two membrane molecules (NOX2 and p22phox), three with a cytosolic distribution (p47phox, p67phox, p40phox), and the G-protein Rac [1] (Figure 1) [33]. The catalytic core of the NOX2 complex is represented by a membrane heterodimer, composed of NOX2 and p22phox (Figure 1), which constitutes the flavocytochrome b558 [38]. It is constitutively expressed in the plasma membrane and in the membrane of specific granule of the neutrophils [39]. In this context, the NOX2 subunit plays a key role in the transfer of electrons from NADPH via FAD and heme to molecular oxygen within the phagosome [40] (Figure 1). NOX2 in humans is encoded by the *CYBB* gene and is composed of two domains, the N-terminal bis-heme cytochrome b, structured in a six a-helical transmembrane segment complexe, and the C-terminal FNR, which contains FAD- and NADPH-binding sites [41–43]. In resting cells, the NADPH oxidase cytosolic subunits, p47phox, p67phox, and p40phox associate in a trimeric complex through specific domains (Figure 1) [44–46]. In the active form of the NOX2 complex, the trimeric complex migrates to the plasma membrane where it interacts with NOX2 and p22phox. p47phox plays a prominent role in the processes of binding and translocation of the cytosolic subunits to the membrane and the subsequent anchoring to p22phox (Figure 1) [47]. In order to prevent ROS overproduction, the protein kinase C- (PKC-) related phosphorylation pathway is implicated in the modulation of these interactions and in particular in the regulation of the interaction between p47phox and p22phox. In particular, in the free and complexed p47phox subunits, the interaction of the autoinhibitory region (amino acids 292-340) with the two SH3 domains prevents its binding to p22phox and, therefore, the NOX2 activation [48]. Upon stimulation, the phosphorylation of the specific serine residues, Ser 303 and 304, activates p47phox, thus allowing the recruitment of p67phox to the trimeric complex within the cytosol (Figure 1) [46, 49]. In turn, p47phox leads to membrane translocation through the binding to p22phox and contributes to the final assembly of the NOX complex that also includes the p22phox [50]. Rac2 and Rap1A, two proteins of the guanine nucleotide-binding proteins family, are also required for the full activation of the NOX complex [51]. Rac2 is located in the cytosol in a dimeric complex with Rho-GDI (guanine nucleotide dissociation inhibitor), while Rap1A is a membrane protein [51, 52]. During the activation, the Rac2-guanosine triphosphate (GTP) complex translocates to the external membrane within the fully assembled multimeric cytosolic complex [53, 54]. The role of Rap1A in the activation of NADPH oxidase is controversial (reviewed in [55]). In fact, Rap1a is expressed at high levels in human neutrophils and copurifies with the p22 subunit of the NADPH oxidase [56–58]. Moreover, activating and inhibitory Rap1 mutants can modify the activity of the phagocyte NADPH oxidase [59, 60]. However, even though cell-free NADPH activation seems to be impaired when the cytosol of neutrophil is depleted of small GTPases and recombinant Rap1A is able to restore NADPH oxidase activation, normal levels of Rap1A have been identified in neutrophil membranes from patients with CGD, suggesting that the absence the cytochrome b558 does not affect the expression of this protein and its presence in the membrane [61]. On the other hand, even though the initial rate of O_2_^−^ in response to formylated peptide and phorbol ester was reduced in *Rap1a−/−* mice, the sustained induction of superoxide production was not significantly reduced, suggesting that unlike Rac2, Rap1A is not necessary for the response of the neutrophil to formylated peptide or phorbol ester [62].

The interaction with the regulatory proteins present in the cytosol is able to induce a conformational change in NOX2 which leads to its activation and to the electron flow. Studies in vitro suggest that the activation of the oxidase may be obtained in the absence of p47phox but not in the absence of p67phox or Rac [63–65]. Moreover, evidence suggests that p67phox plays a pivotal role in the conformational remodelling of NOX2 [66–68]. In particular, the region comprising the residues 199–210 [69] or 190–208 [70] of p67phox has been identified as responsible of the activation of the oxidase.

NOX2 complex plays a key role in killing the microorganisms in phagocytic leukocytes. In particular, the interaction of the external membrane of the phagocytic cell with the bacteria leads to the internalization of a segment of the plasma membrane. This interaction eventually leads to the formation of the intracellular vesicle, where the oxidants O_2_^−^ and O_2_-derived products promote K^+^ influx and induce an increase of the pH within the phagosome [71]. The increase in pH and the K^+^ influx provides an optimal milieu for the activation of the functions of the major granule proteases, elastase, cathepsin G, and proteinase 3 [72]. In fact, the alkaline pH together with the K^+^ influx is able to promote the dissociation of these enzymes from the anionic sulphated proteoglycan matrix to which they are bound.

NOX1 was the first homolog of NOX2 to be described [73]. The *NOX1* gene maps on the X-chromosome both in humans and mice. *NOX1* is highly expressed in the colon epithelium [74]; however, it is also expressed in other cell types, including vascular smooth muscle cells, endothelial cells, and osteoclasts. NOX1 functions in a complex with p22phox [74] and requires two additional proteins, NOXO1 and NOXA1, which are also present in the colon epithelium. NOX1 is activated by the small GTPase Rac, which acts through a direct binding to NOX1 or to the TPR domain of the activator subunit NOXA1. Recent studies in mouse models suggest that NOX1 is implicated in the control of cell proliferation induced by different bacteria, including *Lactobacilli* [75]. NOX1 may also contribute to mucosal repair after injury, as suggested by the evidence of impaired mucosal healing in an intestinal epithelial-specific *Nox1* knockout model [76].

The gene encoding for human NOX3 maps on chromosome 6. *NOX3* is specifically expressed in the inner ear, where it is required for the proper development of otoconia crystals of the vestibular system. It can also be expressed at low levels in the fetal spleen, fetal kidney, skull bone, and brain [77–80]. NOX3 depends on p22phox for its activation [81, 82]. Indeed, the production of ROS by NOX3 is inhibited by truncated p22phox [81]. However, no vestibular dysfunction has been reported for p22phox-deficient CGD patients.

*NOX4* was first identified in kidney epithelial cells, but subsequently, its expression has been detected also in several other cell types, including vascular smooth muscle (VSMC), endothelial cells, cardiomyocytes, skeletal muscle, osteoclasts, neurons, and microglia [33, 83]. Differently from other NOX complexes, the activity of NOX4 does not require cytosolic regulatory subunits even though its activity seems to be enhanced by association with p22phox [70]. Furthermore, NOX4 is the only member of the family which localizes to mitochondria, contributing to mitochondrial ROS levels [84, 85].

*NOX5* is expressed in the spleen testis and endothelial cells [86]. NOX5 oxidase is likely to function as a stand-alone protein since no interactions with any of the known regulatory subunits have been detected. NOX5 oxidase has a unique amino terminal Ca2^+^-binding domain which allows NOX5 oxidase activity to be regulated by increases in cytosolic Ca2^+^ concentration, which could be important in chronic vascular diseases [87].

*DUOX1* and *DUOX2* were initially cloned from porcine and human thyroid glands [88, 89]. However, their expression does not seem to be confined to the thyroid gland. In fact, they are also expressed in the respiratory and gastrointestinal tracts [90–93] where they are involved in the host defence. DUOX1 and DUOX2 differ from the other NOX isoforms because of the homology of the N-terminal domain with the peroxidases [94]. Moreover, NOX5 [95], DUOX1, and DUOX2 [96] are calcium-dependent NADPH oxidase. Finally, while similarly to NOX2, NOX1, NOX3, and NOX5 produce O_2_^−^, which is subsequently converted into H_2_O_2_, NOX4, DUOX1, and DUOX2 are able to directly produce H_2_O_2_. The activity of DUOX is itself regulated by H_2_O_2_ levels [97, 98]. The H_2_O_2_ produced by DUOX2 is able to support the generation of hypothiocyanite, an antimicrobial compound effective against a broad range of bacteria [99–101]. In the thyroid gland, the H_2_O_2_ generated by DUOX2 is necessary to allow the oxidation of the iodide by thyroid peroxidase (TPO) [102]. DUOX2 mutations lead to thyroid dyshormonogenesis, resulting in transient to severe congenital hypothyroidism.

## 3. NADPH Oxidase Deficiency: Model of Inheritance

Mutations in one of the genes encoding the components of the NADPH oxidase complex cause chronic granulomatous disease (CGD), a rare inherited immunodeficiency syndrome with an estimated frequency of 1/200,000 to 1/250,000 newborns. The disorder can be inherited in an X-linked or autosomal recessive (AR) manner and comprises four main genetic forms. The most common form of CGD is the X-linked recessive CGD caused by mutations in the *CYBB* gene, encoding the NOX2 protein. X-CGD represents about 60–70% of the total cases reported to date [103]. The other forms of CGD are AR and are due to mutations in *CYBA*, *NCF1*, and *NCF2*, encoding p22phox, p47phox, and p67phox, respectively [104]. p47phox deficiency is responsible for approximately 30% of AR-CGD, while p22phox and p67phox deficiencies account for the remaining 10% of cases (about 5% each). To date, only one case of AR-CGD due to mutation of the *NCF4* gene, encoding p40phox, has been reported [105]. Different kinds of mutations have been so far reported, including small indel, missense, nonsense, and splice mutations. Missense mutations are frequent in XR-CGD, reducing the *NOX2* expression and functionality in phagocytes at a different extent. The X910 variant identifies the form with null protein and oxidase activity; X91− refers to the form with residual protein expression and reduced, but not absent activity, whereas the X91+ variant is characterized by the normal expression of the protein, which, however, is not functional. Again, in the AR forms, the proteins may be absent (A470, A220, and A670) or normally expressed with residual activity of NADPH oxidase.

Dominant negative *RAC2* mutation has been reported in 2 male infants [106–108]. In the first case, it was associated with soft tissue infections, neutrophilia, and neutrophil chemiotaxis deficiency [106, 107]. The second case was identified in the context of the newborn screening for severe combined immunodeficiency because of a reduction of the T cell receptor excision circles [108]. Apart from the neutrophil dysfunction, this patient also showed leukocytosis, CD4 lymphopenia, and reduced levels of IgA and IgM [108]. More recently, whole exome sequencing revealed a homozygous nonsense loss of function mutation in two siblings with common variable disease, born to consanguineous parents [109]. The clinical phenotype was characterized by recurrent sinopulmonary infections, bronchiectasis, failure to thrive, poststreptococcal glomerulonephritis, coagulation factor XI deficiency, urticaria, food allergy, erythematous plaques, arthralgia, autoimmune thyroiditis, growth hormone deficiency, and hyperparathyroidism. The immunological phenotype included progressive hypogammaglobulinemia, requiring replacement therapy; reduced CD19+ B cells; inverted CD4+/CD8+ ratio; reduced CD4+, CD8+, and recent thymic emigrants; and reduced regulatory T cells. The studies on the neutrophils revealed impaired chemiotaxis, reduced number of neutrophil granules, and morphological changes of the secondary granules. However, neutrophil dysfunction in these patients was not associated with the development of severe clinical manifestations in the neonatal period. The impairment of the B and T cell compartments observed in these patients supports the role of RAC2 in the B and T cell development, also observed in mouse models [110–113].

## 4. Chronic Granulomatous Disease: Clinical Manifestations

CGD is characterized by an increased susceptibility to recurrent or severe infections due to fungal or bacterial pathogens. Infections are typically caused by catalase-positive bacteria and fungi. The most common microorganisms are *Aspergillus* species, *Staphylococcus aureus* (*S. aureus*), *Burkholderia* (*Pseudomonas*) *cepacia complex* (*B. cepacia*), *Serratia marcescens* (*S. marcescens*), and *Nocardia* species [14]. *Salmonella* and *Bacillus Calmette-Guérin* (*BCG*) are common pathogens in patients coming from countries with high prevalence of *Salmonella*, with endemic tuberculosis and mandatory *BCG* vaccine (Table 1). Other rare bacterial species have been recognized in patients with CGD, and the identification of these pathogens is virtually pathognomonic of the disease (Table 1). Among those, it is important to mention sepsis by brackish water organisms *Chromobacterium violaceum* [114] and *Francisella philomiragia* [115]. Another emerging cause of necrotizing lymphadenitis and sepsis are methylotrophs like [116] *Granulibacter bethesdensis* [117, 118], *Acidomonas methanolica* [119], and *Methylobacterium lusitanum* [120]. PCR for 16S rRNA is necessary for the identification of the atypical pathogens [116]. Chronic recurrent pulmonary, hepatic, and cervicofacial infections by catalase-negative actinomycosis have also been reported [121]. Table 1 summarizes the most common and the pathogenetic microorganisms isolated in patients with CGD.

Median age at diagnosis ranges from 2.5 to 3 years. Two thirds of the patients are diagnosed in the first year of life and usually before 5 years of age. The X-linked variants display a more severe clinical picture, accounting for most of the early presentations. On the other hand, AR variants can display milder phenotypes and delayed diagnosis [122–128]. The most common sites of infections are the lungs, lymph nodes, skin, and liver, even though also the bones, kidneys, gastrointestinal tract, and brain may be affected. Presenting features may also include diarrhea, failure to thrive, delayed wound healing, and granuloma formation in multiple organs. Clinical manifestations may be very different among patients.

### 4.1. Lung Involvement in CGD

**Figure 2:**
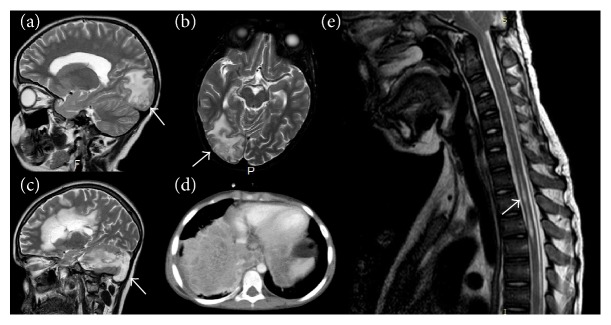
Invasive fungal infections in patients with CGD. (a, b) Cerebral invasion (arrows) in a patient with invasive pulmonary aspergillosis. (c) Cerebellar aspergillosis (arrow). (d) Invasive pulmonary aspergillosis. (e) Spinal cord invasion (arrow) in a patient with pulmonary aspergillosis.

Pulmonary infections are the most common manifestations among all the disease variants. Radiologic findings include consolidation, nodules, areas of scarring, traction bronchiectasis, emphysema, air trapping, mediastinal and hilar lymphadenopathy, pulmonary artery enlargement, and pleural effusion [129]. Pneumonias are recurrent and resistant to standard therapy and can often complicate with granulomatous hilar lymphadenopathy, mimicking sarcoidosis, lung abscesses, and empyema. *S. aureus* was the most common pathogen in lung infections previous to the use of trimethoprim/sulfamethoxazole prophylaxis. In the postprophylaxis era, other bacteria and fungi are the more common causes of pneumonia. *B. cepacia* complex may lead to severe lung infections, bearing a significant morbidity due to the resistance to first-line empiric antibiotic treatments and the delayed diagnosis related to the difficulties in its isolation and growth [130]. Lung infections by *Nocardia* often require invasive techniques for the isolation and the definition of specific antibiotic therapies. In one third of the cases, *Aspergillus* infections are identified along with *Nocardia* infections, probably because both of them are acquired through inhalation [131]. Interestingly, differently from fungal infections, *Nocardia* is able to cause lung cavitations in patients with CGD. In countries with endemic tuberculosis or mandatory *BCG* vaccine, localized or widespread mycobacteria infections are very common, leading to the diagnosis in most of the cases. In a recent survey, mycobacterial infections were identified as responsible for 6% of pneumonias in American patients with CGD [124]. Infections by environmental mycobacteria like *M. leprae* or *M. ulcerans* are uncommon. *BCG* vaccine is contraindicated in patients with CGD [132, 133]. Fungal infections due to inhalation of spores and hyphae are an important cause of morbidity in patients with CGD, and they lead to diagnosis in most of the cases. They may present with insidious and subclinical courses, with aspecific symptoms like growth failure and asthenia, low-grade fever, cough or chest pain, and mild leukocytosis. On the other hand, the inhalation of aerosolized decayed organic matter, including fungi, can cause an acute fulminant pneumonitis also known as “mulch pneumonitis” [134]. Lung infections are typically caused by *Aspergillus* species (Figure 2(a)), and *A. fumigatus* is the most common [135]. Other variants like *A. nidulans* [136], *A. viridinutans*, and *Neosartorya udagawae* [137] are almost pathognomonic for CGD and can cause invasive spreading disease to the bones and nervous system [138]. Other fungal pathogens isolated in patients with CGD include molds *Paecilomyces lilacinus* [139] and *variotii* [140], *Geosmithia argillacea* [141–143], the fungus *Penicillium piceum* [144], and the basidiomycetes of the *Phellinus* species [145]. The recognition of atypical fungal infections can be challenging due to the need for nonstandard culture conditions. For this reason, extensive and aggressive diagnostic approaches, including molecular diagnostic, should be always undertaken in order to identify the pathogens, especially when the patient is under mold prophylaxis [138, 146]. Yeast infections are less common in patients with CGD. *Mucormycosis* has been only reported in the setting of immunosuppression [137], and dimorphic mold infections like *Coccidioidomycosis* and *Blastomycosis* are not typical [147].

### 4.2. The Skin and Lymph Nodes

Cutaneous involvement with abscesses and deep seeded infections is also common. The pathogens implicated are typically *S. aureus*, catalase-positive and gram-negative bacteria. Cutaneous abscesses may often require prolonged antibiotic courses, incision, and drainage procedures for resolution. Impetigo in the nasal area and resistant facial acne caused by *S. aureus* also require long courses of local and systemic antibiotics [148–154]. Suppurative adenitis due to *S. aureus* has been reported. *Granulibacter bethesdensis* is an environmental organism that can lead to necrotizing pyogranulomatous lymphadenitis and in rare cases fatal bacteremia. Noninfectious manifestations of CGD include photosensitivity, discoid lupus, granulomatous lesions, and vasculitis. Patients with CGD display typically delayed and abnormal wound healing caused by excessive granulation, causing scarring and wound dehiscence [151].

### 4.3. The Liver

Hepatic and perihepatic abscesses are common in CGD. Liver involvement may lead to morbidity due to the challenging diagnosis and treatment. Presentation can be subtle, with aspecific systemic symptoms. Differently from hepatic abscesses in immunocompetent patients, commonly caused by enteric bacteria, abscesses in CGD are mainly caused by staphylococci. These abscesses are usually very dense and may require difficult surgical procedures in most of the cases. Recently, the association of high-dose steroids and antibiotic regimens has been proved to be an effective surgical sparing approach [155, 156]. Noninfectious causes of liver involvement in patients with CGD include toxic drug-induced hepatitis, hepatosplenomegaly due to portal venopathy, and nodular regenerative hyperplasia. The development of noncirrhotic portal hypertension is a negative prognostic factor [157, 158].

### 4.4. Metastatic Infections

Osteomyelitis is one of the most important metastatic infections in patients with CGD. It is characteristically multifocal, with other organs involved, caused by typical CGD pathogens like *S. marcescens* or *Aspergillus* species. Biopsy reveals signs of chronic inflammation and granulomatous features [159, 160]. Infection spreading can also affect the central nervous system, presenting with meningitis, brain, and epidural abscesses (Figures 2(b)–2(e)) [148–150]. Genitourinary tract involvement is also frequent (38%), but often occult. Granulomatous cystitis, ureteral and urethral obstruction, prostate abscesses, bladder granulomata, and urinary tract infections have been reported [161–164]. Chorioretinal lesions are relatively common in patients with CGD, typically with “punched out” lesions associated with pigment clumping lying in line with the retinal arteries. Those lesions appear to be nonprogressive and usually do not impair vision [165]. Since bacterial DNA was isolated from those lesions, infections may play a role in the pathogenesis [166]. However, these lesions in most cases do not progress during immunosuppression suggesting that they are not sites of active infections [122]. Patients can also present with blepharokeratoconjunctivitis and pannus formation [167].

### 4.5. The Gastrointestinal Tract

The gastrointestinal tract is extensively affected in patients with CGD. Granulomatous disease-mimicking Crohn's disease, affecting the oesophagus, stomach, jejunum, ileum, cecum, and rectum, is common [8, 152–154, 168]. Also, the upper gastrointestinal tract may be involved in the forms of gingivitis and gingival hypertrophy, stomatitis, and aphthosis. Around a third of patients are affected, being more frequent in the X-linked forms (43%) then in the autosomal recessive (11%). In some cases, it may represent the only manifestation of the disease. In such cases, diagnosis may be challenging [169]. Clinical presentation is variable, ranging from gastric outlet obstruction [170], malabsorption, vitamin B12 deficiency to classic inflammatory bowel disease symptoms like abdominal pain, diarrhea, fistulae, and strictures. CGD-related colitis is often resistant to the standard inflammatory bowel disease therapies. The treatment with anti-TNF-*α* drugs has been successfully used to treat fistulas, but it may increase the risk of severe infections [171].

### 4.6. Brain Disease

The assessment of cognitive function in 23 patients with CGD revealed an increased prevalence of cognitive deficit (IQ < 70%) [172]. However, this evidence was not confirmed in a subsequent study [173]. It should be noted that in both the studies, the sample size was quite small to draw definitive conclusions. Considering the role of superoxide generation in brain hippocampal synaptic plasticity and hippocampus-dependent memory, this subject deserves further studies [174]. On the other hand, increased levels of NOX2 have been detected in microglia and infiltrating macrophages in brain autopsies from patients with initial multiple sclerosis, suggesting a role of ROS in the pathogenesis of demyelination and neurodegeneration [175]. This hypothesis has been confirmed by the evidence of the involvement of NOX2 in motor neuron degeneration in a mouse model of amyotrophic lateral sclerosis [176].

### 4.7. Growth Defect

Children with CGD usually attain a height within their target by adulthood, despite being generally small for their age in early childhood. Growth failure might be linked to colitis and malabsorption and to the chronic and frequent infections [177]. Regardless of the cause, hematopoietic cell transplantation is able to reverse most of the cases of growth defect [178].

### 4.8. Laboratory Findings

Patients with CGD may show a mild to moderate leukocytosis, elevation of acute phase reactants, and polyclonal hypergammaglobulinemia. A chronic microcytic anemia due to chronic disease and iron deficiency may be observed at the onset of the disease, especially in association with colitis. Patients can also display a T CD4+ lymphopenia [179, 180].

### 4.9. McLeod Phenotype

The *CYBB* gene, responsible of the X-linked CGD variant, is located at the Xp21.1 locus. Deletions across this locus can also involve adjacent genes, generating complex phenotypes. Telomerically to the X-CGD locus, there are the Kell erythrocyte antigens. Their deletion causes the McLeod phenotype, a syndrome with haemolytic anemia associated with neuroacanthocytosis [181, 182]. For this reason, patients with X-linked CGD need to be carefully evaluated for their Kell phenotype in order to avoid sensitization and transfusion reactions [183]. Larger telomeric deletions can also involve the retinitis pigmentosa GTPase regulator (*RPGR*) gene, responsible for X-linked retinitis pigmentosa, and *DMD*, causing Duchenne muscular dystrophy. Large centromeric deletions can involve the *OTC* gene, causing ornithine transcarbamylase deficiency [184].

## 5. Williams Syndrome

Williams syndrome (WS) is a rare genetic disorder, affecting 1/20000 newborns, characterized by neurodevelopmental alterations leading to mild-to-moderate mental retardation and characteristic craniofacial features. Supravalvular aortic stenosis and hypercalcemia in infancy may also feature the disease. WS is due to a heterozygous segmental microdeletion (about 1.5–1.8 Mb) at chromosomal band 7q11.23. This deletion involves about 20 genes and, in some cases, it may also include the *NCF1* gene. In extremely rare cases, the WS microdeletion may be associated with a mutation in *NCF1* on the other allele. When the WS deletion includes *NCF1*, this combination leads to the development of CGD [185, 186].

## 6. NADPH Oxidase Deficiency and Autoimmunity: A Focus on Female Carriers and Patients Carrying Oxidase Gene Polymorphisms

Autoinflammatory and autoimmune disorders have been identified in patients with CGD, female carriers for NOX2 deficiency, and patients carrying oxidase gene polymorphisms, suggesting a role for NADPH oxidase in the pathogenesis of autoimmunity and in the in regulation of the adaptive immune responses [187–189]. In a recent study, Wen et al. showed that NOX2 is critical for the correct functioning of the suppressive machinery of CD8+ T regulatory cells (CD8 Treg) [190]. In particular, CD8 Treg are able to control the intensity of effector T cell responses by releasing NOX2-containing microvesicles which are in turn taken up by the target cells [190]. NOX2-derived ROS abrogate the phosphorylation of ZAP70 and LAT preventing the activation of the effector cell [190]. This mechanism is impaired in older individuals and is related to the development of autoimmunity [190]. Finally, *NOX2* overexpression is able to restore the suppressive function of CD8 Tregs from older donors [190].

Genome-wide association studies (GWAS) have revealed the association between genes-encoding oxidase subunits and autoinflammatory and autoimmune disorders. The most common autoimmune manifestations in CGD are systemic lupus erythematosus (SLE) [124–126, 191, 192], followed by thrombocytopenic purpura (ITP) [124–126, 191, 193–195] and arthritis [125, 192, 193, 196]. Variations in *NCF2*, encoding p67phox, and *NCF4*, encoding p40phox, have been associated with SLE [197] and with rheumatoid arthritis [198] and Crohn's disease [199, 200], respectively. Moreover, a recent study revealed that p47phox-deficient patients are at greater risk of developing severe diabetes and the related complications, including renal and cardiovascular disease, as compared with patients with NOX2 deficiency [201]. Similarly, GWAS studies revealed variants in NOX2 complex components in patients suffering from very early onset inflammatory bowel disease (VEOIBD) [202–204]. Apart from NOX2, also other NOX homologues seem to play a role in the development of VEOIBD. In a recent study, two variants of *NOX1* have been identified in three patients with severe pancolitis [205]. The first patient carried a missense variant located upstream of the first FAD-binding domain. This mutation lead to a reduction of ROS generation by 50–60% [205]. The second variant was located in the second FAD-binding domain and led to a 60–80% decrease in ROS production. Similarly, a *DUOX2* missense variant was identified in a patient with recurrent pancolitis, complicated by perforation and colectomy. The variant was located in the third intracellular loop and leads to reduced H_2_O_2_ generation [205]. A second *DUOX* variant located in the highly conserved GRP sequence in the third NADPH-binding domain was identified in a patient with pancolitis [205]. This variant also led to decrease in H_2_O_2_ release [205].

**Figure 3:**
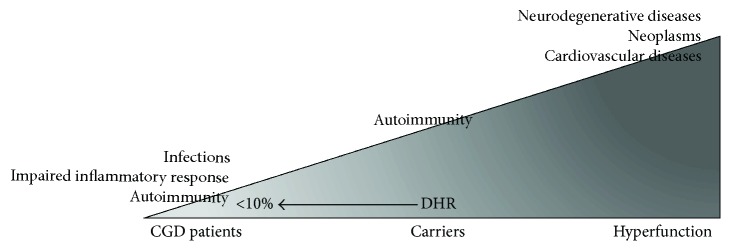
Spectrum of clinical manifestations associated with marked reduction (dihydrorhodamine (DHR) < 10%), slight reduction, and increase of the NOX2 activity. The complete absence of NOX2 activity leads to the development of infectious, inflammatory and autoimmune complications observed in CGD. A partial reduction of the NOX2 activity, observed in female carriers, may lead to the development of autoimmune complications. Upregulation of NOX2 has been observed in different cardiovascular and neurodegenerative disorders and in neoplasms.

Female carriers for NOX2 deficiency are not completely asymptomatic. In affected women, lyonization determines two populations of phagocytes. One with normal oxidative activity and another with impaired oxidative activity. Usually, 15 to 20 percent of wild-type cells are sufficient to handle infections. However, in case of unbalanced lyonization leading to less than 20 percent of normal oxidase activity, female subjects can show mild to severe CGD phenotype (Figure 3). Skewing of X-chromosome inactivation can be progressive with aging leading to late onset CGD manifestations and increased rate of autoimmunity including inflammatory bowel disease [206–209]. The most common findings in heterozygous female carriers are photosensitive skin rashes (58%), mouth ulcers (42%), and joint pain (37%). The cutaneous rash closely resembles chronic discoid lupus, even though systemic lupus serologic markers are usually negative (Figure 3) [210–212]. Asymptomatic carriers can also have the typical chorioretinal lesions. All the abovementioned manifestations seem to be associated with skewed lyonization [165, 167]. In a few cases, a severe phenotype, requiring HSCT, has been described in X-CGD female with nonrandom X-chromosome inactivation [213].

Both the X-CGD carrier status and X-chromosome inactivation can be identified with the dihydrorhodamine (DHR) 123 testing. Recently, Marciano et al. studied a large cohort of X-linked CGD female comparing the presence of infections and/or autoinflammatory manifestations with the levels of DHR in the peripheral blood neutrophil. Overall, 48% of X-CGD carrier had a clinical history of infections and/or autoimmune or inflammatory manifestations. A significant correlation was found between the development of infections and levels of DHR lower than 20%. Furthermore, the authors found that carriers more prone to develop severe infections had DHR lower than 10%. No correlation was found between different *NOX2* mutations and % of DHR values. However, differently from what was observed for the infections, it was not possible to find any relationship between the DHR levels and the incidence of autoimmune or inflammatory manifestations. These data support the hypothesis that, differently from infectious complications, the development of inflammatory manifestations is not correlated with the residual reactive oxygen intermediate production but to the carrier status per se. Further factors, including the influence of confounder genes, and environmental factors such as the microbiome may contribute to the development of these manifestations [191, 214]. Considering that the X inactivation can be different between tissues, further studies are necessary to define the role of X inactivation in the pathogenesis of inflammatory or autoimmune manifestations in X-CGD carriers.

## 7. NADPH-Dependent ROS Deficiency and Autophagic Dysfunction: Implication for Infectious Response and Inflammasome Activation

Autophagy is implicated in many tissue-specific functions and cellular pathways, including those involved in both innate and adaptive immunity [215, 216]. Autophagy is a fundamental metabolic pathway implicated in delivering cytoplasmic proteins and organelles to the lysosome for degradation. Recent evidence suggests that autophagy is also implicated in innate immune response pathways and in particular in targeting intracellular bacteria in the cytosol and in limiting bacterial growth in damaged vacuoles and phagosomes. The process, also known as “xenophagy,” involves the formation of double-membrane compartments, namely, autophagosomes, around target bacteria and their transport to lysosomes, where they are degraded [217, 218]. In particular, this mechanism seems to be mainly implicated in the defence against intracellular infectious agents, including *Mycobacterium tuberculosis*, *Salmonella*, *Shigella*, *Legionella*, *Burkholderia* species, and *Aspergillus fumigatus* [219–221]. Most of these infectious agents are usually responsible for fatal or severe infections in patients affected with CGD. On the other hand, bacteria have developed several mechanisms to impair autophagy interfering with autophagy signalling or the autophagy machinery [222]. The evidence that the LC3 (light chain 3) protein, a member of the autophagy-related family (Atg) and beclin 1, another autophagic component, is able, upon bacterial cellular engulfment, to bind to the phagosomes promoting their fusion with lysosomes and the killing of the enclosed microbes suggested that autophagy may be implicated in antimicrobial defence [223]. This process has been found strongly decreased in phagocytes from *Nox2* KO mice [224]. Furthermore, it has been observed that in both human and murine models of CGD macrophages, which explain a permissive phenotype for bacterial replication, autophagy induction by rapamycin, a mammalian target of rapamycin (mTOR) inhibitor, is able to reduce *B. cenocepacia* bacterial burden, suggesting that the increase of the autophagic flux may represent a potential therapeutic approach to improve bacterial clearance in CGD [219]. A normal ROS production is considered indispensable for the physiological activation of the autophagic process [225]. On the other hand, the integrity of the autophagic machinery results in the inhibition of IL-1*β* production. In a recent study, ROS deficiency has been associated with defects of autophagic mechanism in both mice and patients with CGD. In this study, the human subunit p47phox was showed to be fundamental for LC3 recruitment after the bacterial internalization. Moreover, it was observed that IL-1*β* production from human CGD monocytes was significantly increased as compared to normal controls. The blocking of the IL-1 receptor with anakinra resulted in a significant reduction of the inflammation in *p47phox−/−* mice affected with colitis associated with a reduced production of inflammatory cytokine. A clinical improvement of the colitis and perirectal abscesses was, also, observed in two CGD patients affected with active colitis, treated with the same drug [15]. Unfortunately, anakinra did not induce a clinical remission in further five cases [14]. According to the indication of the European Medicines Agency, the dose of anakinra may be gradually increased to a maximum of 8 mg/kg/day, based on the individual therapeutic response (http://www.ema.europa.eu/docs/en_GB/document_library/EPAR_-_Product_Information/human/000363/WC500042310.pdf). Since in the study by Hahn et al. the protocol of treatment and exact dose of drug used per patient are not specified, it is not possible to rule out that the dose of drug used was too low. The treatment with anakinra was shown to be able to modulate the increased inflammasome activation and to restore the abnormal autophagy in both human and mouse cells. In CGD mice infected with *Aspergillus fumigatus*, the treatment with anakinra was also associated with a reduction of fungal growth and granuloma formation resulting in an increased survival. This last evidence suggests that the pharmacological restoration of the autophagy may be also useful in treating severe fungal infection. In a further study by Gabrion et al. [17], rapamycin was able to induce a reversion of CGD inflammatory status in different immune cells of patients with CGD. In particular, rapamycin was able to induce a reduction of the production of IL-1*β*, IL-6, IL-23, and TNF-alpha in macrophage stimulated with LPS, a reduction of the IL-6/IL-10 proinflammatory ratio, and a decrease of the inflammasome activation. Moreover, the addition of low doses of anakinra was able to potentiate the inhibitory effect of rapamycin on IL-1*β* secretion in vitro. Therefore, drugs that modulate autophagy may represent a potential therapy for both infectious and inflammatory manifestations associated to this complex congenital disorder.

## 8. NADPH Oxidase Upregulation and Vascular Disease

Apart from the phagocytes, where it has been originally identified, *NOX2* is also expressed in endothelial cells [18], cardiomyocytes [19], hematopoietic stem cells [20, 21], and platelets [22]. *NOX2* upregulation has been shown to be involved in neurodegenerative diseases [23], neoplasms [24], and cardiovascular diseases [25–28]. NOX can exert damaging as well as protective roles in the vascular system. In fact, while a regulated production of H_2_O_2_ is necessary to maintain the integrity of the endothelial system and to control the inflammatory response [226], on the other hand, the hyperproduction of H_2_O_2_ may lead to inflammation, oxidative stress, and endothelial disfunction [227–229]. Among the different vascular NADPH oxidase isoforms, NOX2 is the most widely expressed and it has been identified in VSMCs, adventitial fibroblasts, endothelial cells, and perivascular adipocytes [18, 230, 231]. In the cells of the vascular system, the structure of NOX2 complex is similar to that found in phagocytes. However, in some conditions, NOX organizer protein 1 (NOXO1) and NOX activator protein 1 (NOXA1) may play a role in the activation of NOX2 complex [232]. NOX2 is implicated in proliferation and vascular endothelial growth factor- (VEGF-) induced migration [233] and in the regulation of the expression of adhesion molecules during inflammation and angiogenesis.

Recent studies suggest that NOX2 activation is involved in atherosclerosis [234–236]. Several experimental studies showed an upregulation of the *NOX2* in the atheroma from carotid [237–239] and coronary sites [240, 241]. In an experimental model of carotid lesion induced by flow cessation, the overexpression of the NOX subunit *p22phox* led to a progression of carotid artery lesions, which was more marked compared to lesions in wild-type mice [242]. In atherosclerotic coronary arteries, *p22phox* was overexpressed in the neointimal and medial smooth cells and in infiltrating macrophages in hypercellular regions at the border of atheromatous plaques [243]. Furthermore, enhanced superoxide production was detected in coronary arteries from patients with coronary heart disease in association with upregulation of *p22phox* and *NOX2* suggesting that both these subunits contribute to oxidative stress in human coronary atherosclerotic lesions [244]. Endothelial cells produce O_2_^−^ prevalently via different NOX isoforms such as NOX1, NOX2, NOX4, and NOX5, which may contribute to modulate arterial dilatation with different mechanisms [245, 246]. Flow-mediated dilatation (FMD), which is dependent upon endothelial release of NO and is considered a surrogate marker of atherosclerosis [29], has been evaluated in patients with NOX2 (X-linked CGD) [30] or p47phox hereditary deficiency. Patients with NOX2 and p47phox hereditary deficiency showed enhanced FMD, which, however, was more marked in NOX2-deficient patients suggesting a relationship between rate of ROS formation and artery vasoconstriction [30–32]. In accordance with these studies, compared to controls, higher values of FMD were found in carriers of hereditary deficiency of NOX2, suggesting that also a partial reduction of NOX2 can contribute to increase endothelial function [247]. The mechanism accounting for enhanced artery dilatation was attributed to heightened NO generation, which was also suggested to account for enhanced vasodilation detected in animal knockout for *NOX2* [248, 249]. The evidence that the intravenous injection of L-NAME, an inhibitor of NO synthase, is able to blunt the increase of the FMD in patients with X-linked CGD [32] also confirms this hypothesis. The close relationship between endothelial dysfunction and NOX2 has been also studied in other models including dyslipidemia, obesity, smoking [250, 251], hypertension, metabolic syndrome, diabetes mellitus, peripheral arterial disease [252], and obstructive sleep apnea [253]. These classic cardiovascular risk factors provoke endothelial dysfunction even in childhood, and the coexistence of NOX2 regulation suggests this enzyme as a potential trigger [254–256]. In fact, children with hypercholesterolemia, obesity, or obstructive sleep apnea displayed *NOX2* upregulation coincidentally with a reduced FMD [30, 254–256]. A significant reduction of carotid intima-media thickness (IMT) [30], which is a surrogate marker of atherosclerosis, has been detected by Doppler ultrasonography [257] in children [30] with CGD and later confirmed in an adult cohort of female carriers with NOX2 deficiency [247]. The same evidence was then confirmed using a more sophisticated diagnostic approach, that is, magnetic resonance imaging and computed tomography, which showed that CGD patients, compared with control subjects, had a 22% lower internal carotid artery wall volume with a similar reduction detected in both the p47phox- and NOX2-deficient subtypes [258]. Based on the above-reported experimental and clinical data, NOX2 could be suggested as potential target to counteract the process of atherothrombosis; however, interventional studies with NOX2 inhibitors are warranted to assess the clinical validity of this therapeutic approach in patients at risk or with cardiovascular events. Apart from NOX2, also NOX4, NOX5, p22phox, and, to a lesser extent, NOX1 may be implicated in the pathogenesis of atherosclerosis [234, 259]. In particular, similarly to NOX2, NOX1 seems to be implicated in the acute response to injury or to angiotensin II stimulation. On the contrary, NOX4 seems to be able to maintain VSMC in a quiescent status [237]. For this reason, the association of high levels of NOX2 and low levels of NOX4 may lead to the development of intimal hyperplasia, remodelling, and accelerated atherosclerosis in vein grafts [260]. NOX5 is also overexpressed in coronary artery obtained from patients with coronary artery disease [261]. The increase of NADPH oxidase activity may also contribute to the development of atherosclerosis through lipid oxidation.

NADPH oxidase is also implicated in the pathogenesis of hypertension. Angiotensin II, which plays a critical role in the pathogenesis of hypertension, is able to induce the expression of different NOX homologues, including *NOX1*, *NOX2*, *NOX4*, and *p22phox* [26, 233, 262]. The induction of the NOX by angiotensin II increases the production of superoxide and H_2_O_2_ in VSMC, also leading to vascular hypertrophy. In a mouse model of p47phox deficiency, angiotensin II was not able to induce an increase of the production of O_2_^−^, different from what was observed in wild-type mouse [263] suggesting that p47phox deficiency is able to attenuate angiotensin II-induced hypertension [264] and endothelial dysfunction [265]. Deletion of p47phox was also associated with the prevention of angiotensin II-induced aneurysm formation [266–268] and myocardial infarction-induced cardiac dysfunction [263]. p47phox deficiency is able to impair both NOX1 and NOX2 activation. Overexpression in VSMC of *NOX1* and *p22phox* in mouse has been associated with elevation of blood pressure in response to angiotensin II and with the development of vascular hypertrophy [269–271]. On the other hand, the deletion of both the subunit has a protective role against the development of endothelial dysfunction, oxidative stress, vascular hypertrophy, and aortic dissection [248, 272–274]. NOX2 is implicated in the development of vascular hypertrophy [275] and endothelial dysfunction [276], but it does not seem to be directly implicated in the regulation of blood pressure [275]. *NOX4* overexpression in endothelial cells is associated with vasodilatation and reduced blood pressure, suggesting that it has a protective role in hypertension and the related complications [277]. Evidence suggest that NOX4 may have a protective role in ischemic and hypertensive stress [278]. However, studies in the brain suggest that NOX4 contributes to the oxidative stress in the context of stoke or other pathologic conditions [279].

NOX1 and NOX4 are also implicated in the pathogenesis of long-term macrovascular complications in diabetic patients [280]. Hyperglycaemia is able to directly induce the NADPH oxidase leading to increased ROS production and eventually to endothelial dysfunction [281].

Vascular NADPH oxidase also plays an important role in tumour growth [282] and ischemic revascularization [283]. In particular, NOX2 is implicated in endothelial cell migration [283] and NOX1, NOX4, and NOX5 are implicated in angiogenesis and neovascularization [284]. NADPH oxidase may be activated by different proangiogenic factors [285], including VEGF [286], and in turn can modulate the release of different angiogenesis-related factors, including VEGF-A [287], hypoxia-induced factor 1a (HIF-1a) [288], and matrix metalloproteinases (MMPs) [289, 290].

In pulmonary artery smooth muscle cells (PASMCs), NOX2 and NOX4 are involved in the development of pulmonary hypertension in response to chronic hypoxia [291, 292]. In particular, in response to hypoxia, the ROS produced by NOX2 activate NOX4 which in turn is able to bind HIF-1*α*, leading to PASMC proliferation [285, 292].

Finally, NOX1, NOX2, and NOX5 are also implicated in the regulation of the expression of the endothelial adhesion molecules, including vascular cell adhesion molecule 1 (VCAM-1), intercellular adhesion molecule 1 (ICAM-1), and P- and E-selectins. The increased expression of these molecules leads to transmigration of leukocytes into the subendothelial space [293] which is implicated in early atherosclerosis [294–296] or hypertensive vascular dysfunction [297].

## 9. Hematopoietic Stem Cell Transplantation for the Treatment of CGD

**Figure 4:**
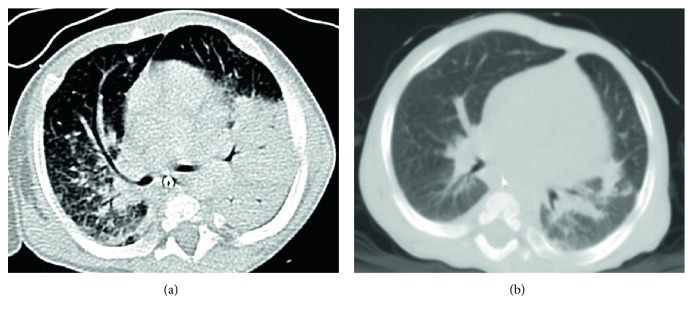
CT scan of a possible invasive fungal infection in a 5-month-old XCGD patient (a) pre-HSCT and (b) after HSCT. A progressive improvement of the areas of consolidation in the left and right lungs, especially for the right lobes, is observed. Residual pulmonary lesions are visible in the left and right inferior lobe.

To date, hematopoietic stem cell transplantation (HSCT) represents the only available potentially curative treatment for patients with CGD of any genetic origin. In the last decade, improvements in HSCT protocols led to a significant improvement of the survival of patients with X-linked CGD (Figure 4). Currently, the two most common conditioning regimens include reduced intensity conditioning (RIC), typically busulfan based or a reduced toxicity regimen (typically treosulfan based). Both of them have resulted in an extremely improved survival (>90%) [298–301]. These encouraging results corroborate the priority to extend the HSCT indications. In 2014, Güngör et al. published the largest prospective study of a reduced intensity conditioning regimen for HSCT in pediatric and adult patients with CGD [300]. In this study, HSCT was only considered in patients with more than one life-threatening infection, no compliance with antimicrobial prophylaxis, or steroid-dependent autoinflammation [300]. However, other studies showed an impressive lower survival rate in patients with X-linked CGD treated conventionally with antibiotic and antifungal prophylaxis as compared with patients treated with HSCT [302]. In fact, patients surviving childhood face a high mortality due to a cumulative organ injury that could also dramatically affect HSCT outcome or even preclude this treatment [303]. For this reason, current clinical management of these fragile patients suggests that HSCT should be considered as a reasonable curative treatment for a wider group of patients as soon as possible and, when possible, before the onset of severe complications [302]. Apart from preexisting comorbidities and organ dysfunction, standard myeloablative conditioning protocols were also responsible for the striking transplant-related mortality observed in the past. Granulocyte infusions and more recently the use of pioglitazone, that can bypass the inability of NADPH oxidase complex by increasing mitochondrial reactive oxygen species, suggest relevant insights in the treatment of this rare disease [304, 305].

## 10. Gene Therapy for the Treatment of X-Linked CGD

The evidence of the high success rate of early HSCT in patients with CGD [201, 300, 306, 307] has opened the way to gene therapy (GT) approach for patients without a matched donor. A protocol based on *γ* retrovirus (*γ*RV) GT without conditioning was developed and resulted in transitory functional correction of ≤0.5% of peripheral blood granulocytes, without any long-term clinical benefit [308, 309]. The main reasons of the failure of this approach were the lack of survival advantage of the transduced neutrophils over the defective ones, their short lifespan, and the inflammatory bone marrow milieu, with a potential negative effect on HSC gene transfer [310–312]. Subsequent trials with *γ*RV vector-transduced, mobilized CD34+ cells were attempted between 2000 and 2010 [306, 311, 313, 314]. However, in most of the cases, a loss of the long-term engraftment of transduced cells [315] was observed. Moreover, this approach was also complicated by the occurrence of myelodysplasia and clonal expansion in both adult and pediatric patients. The safety concern led to the clinical use of regulated SIN-lentiviral vectors targeting *NOX2* expression in myeloid cells. In vitro and in vivo murine studies showed that this vector is able to restore NADPH oxidase activity and has a safer profile [316]. This vector is currently employed in multicenter trials in Europe and in the USA (NCT01906541, NCT01855685, and NCT02234934).

## 11. Conclusions and Perspective

Loss-of-function mutations in genes encoding for various NADPH oxidase subunits lead to a phenotype mainly characterized by severe and recurrent infections. However, recent evidence shows that NADPH oxidase is also implicated in the regulation of several diverse mechanisms. The aim of this review is to focus on the most recent evidence regarding the biological role of NADPH oxidase and its implication in the typical CGD clinical manifestations, as well as in the more recently defined clinical features, including autoinflammatory manifestations. The identification of the pathogenic mechanisms underlying inflammatory complications has led to the identification of novel potential curative and symptomatic approaches, whose effectiveness *in vitro* and *in vivo* has been explored in some preliminary models and clinical trials. New biological roles of NADPH oxidase are emerging, that is, the implication in the pathogenesis of atherosclerosis. These observations are paving the way for future observational and interventional studies on CGD-affected patients and carriers.

## Figures and Tables

**Table 1:** Microorganisms causing infections in CGD

Common	Reference
*Aspergillus* species	[14]
*Staphylococcus aureus*	
*Burkholderia cepacia complex*	
*Serratia marcescens*	
*Nocardia*	
*Salmonella*	
*Bacillus Calmette-Guérin* (*BCG*)	
Pathognomonic	
*Chromobacterium violaceum*	[114]
*Francisella philomiragia*	[115]
*Granulibacter bethesdensis*,	[117, 118]
*Acidomonas methanolica*	[119]
*Methylobacterium lusitanum*	[120]
*Actinomycosis*	[121]
*A. nidulans* and *viridinutans*	[136]
*Neosartorya udagawae*	[137]
*Paecilomyces lilacinus* and *variotii*	[139, 140]
*Geosmithia argillacea*	[141–143]
*Penicillium piceum*	[144]
*Phellinus* species	[145]
